# Extracts and Gleanings

**Published:** 1876-05

**Authors:** 


### Extracts and Gleanings.

---

*Ulcers*—Are treated at Charity Hospital, N. Y., as follows: A general principle of treatment adopted in every case, with a single exception, that day, was that of strapping with adhesive plaster and using a firm roller bandage. Each ulcer was snugly strapped with either transverse or basket strapping, and the limb firmly bandaged.

The dressing for the ulcers was varied according to the requiremeuts of each case, certain appearances indicating the use of certain applications. Some experience is requisite to recognize the exact condition requiring the use of a certain remedy, which, if properly applied—applied in strict conformity to the indication—renders the general treatment much more satisfactory.

Examples of specific ulcers are somewhat numerous here. One was dressed with iodoform and glycerine (sat. sol.) before applying the straps. It required a little stimulating.

For all these cases of gangrenous and sloughing ulcers either a saturated solution of carbolic acid is used, or a solution of bromine, grs. xvi. to the ounce of water.

The bromine is the more common application, and it most admirably rids the surface of that sloughy, nasty condition invariably present in such cases.

Traumatic ulcers generally receive the extra attention of scraping epidermal scales from the leg with a pocket-knife and spreading them over the surface of the ulcer. Since this has been practised, the ulcer has healed much more rapidly than before, with no different treatment otherwise. Over these scales was placed a dressing of balsam of Peru, and over this the straps and bandage as usual. Covering the surface of the ulcer with a coating of balsam of Peru is the ordinary method of dressing all ulcers that require no special application, and it is quite generally used in connection with the special application. For example, balsam of Peru and iodoform (in powder) act together better than either alone. One is soothing and the other stimulating, and the joint benefit received from the combination is much greater than that received from either alone. When ulcers are covered with exuberant granulation, the solid stick of nitrate of silver is applied, and over this the ordinary dressing.

Ulcers with thickened edges are reduced by freely cutting through the indurated mass in transverse incisions throughout the entire circumference of the ulcer.

The treatment of varicose ulcers forms no exception to the general rule.

A man who had both legs covered with ulcers, from half an inch to an inch in diameter, resulting from hard service and low salt diet at sea, received the iodoform dressing, the ulcers being neither strapped nor bandaged.

An old man in bed with an ulcer involving about two-thirds of the circumference of his leg, which had been in an extremely sluggish condition, now exhibits a lively growth of healthy-looking granulations all over its surface, which had been established within three or four days by the use of the following:

| | | |
|---|---|---|
| R | Flour | oz. iv. |
| | Gum tragacanth | oz. ss. |
| | Gum arabic | oz. j. |
| | Egg, No | i. |
| | Chalk | drchm. iij. |

Make a paste by adding boiling water, and while fresh place it on the ulcer with a brush, four times a day. When the material sours it must be changed.

One great advantage derived from this general plan of treatment by strapping and bandaging is, that the patient can go about without detriment to the ulcer.

*Burns.*—The chief agents employed in the treatment of this class of difficulties in Charity Hospital, N. Y., are the carbolic salve and Carron oil.

| | | |
|---|---|---|
| R. | Lime water | oz. viij. |
| | Flaxseed oil | vij. |

In some cases a little turpentine is added to the Carron oil.

*Diseases of the Uterus.*—Dr. H. T. Cleaver, in reporting cases for the Iowa Medical Society, said: "I regard the maintained position of the organ as a condition essential to success, and that instead of resorting to the means so commonly used in modifying the function of nutrition in a hypertrophied cervix by the employment of the sacrificator, leeches, blisters, the cautery, or caustics, the same end is accomplished by temporary aid to the natural supports, and modifying the supply of the elements of nutrition by irrigating the parts with the medicated injections. I do not say that it is possible to dispense entirely with the implements of the heroic practice by discarding the knife, the cautery, or the numerous caustic preparations, but I do say that from an extensive employment of these remedies in years gone by, I have not that reverence for them that I once had, nor have I realized those benefits which seem to have attended their use by others; but on the other hand I have realized those good effects from the employment of means less objectionable to the patient, and more speedily and surely accomplished a cure among the multitude of means that have been used for the relief and cure of the uterine displacements and their complications. I know of none that have in any degree met the requirements so nearly as the various forms of the Hodge or level pessary. There are others; the ring of Meigs and the spring of Scattergood that possess value in particular cases, but do not cover that wide range of applicability that characterizes the Hodge instrument. With it I have relieved cases that have apparently been amenable to no other known appliance, and in numerous cases radical cures have been realized where a palliative effect was only anticipated. The cases selected to illustrate the value of this curative as well as palliative implement are by no means isolated ones. That cases present themselves that prove rebellious to not only this, but every other known means of relief, is a matter of fact patent in the experience of every man who has had an extended practice in this department of professional business. I do not desire to bestow upon this or the other means employed in the cases cited, unmerited praise, but I do desire that the burning, torturing plan, so long pursued in the treatment of diseases of this organ, be dispensed with where means less repugnant to the patient, less troublesome to the practitioner, and more successful in results, may be tried.

By the method suggested by Scanzoni, of irrigating with medicated or plain water, many of the inflammatory affections of the uterus and its appendages have, under my observation, yielded speedily, after resisting for months and years other methods of treatment.

As a local application to the diseased surface in that particular form of cervical endometritis, specified in the case reported, I regard the finely powdered per sulph. of iron as of more value than any other of which I have had experience. Two or three applications at intervals of six or eight days, and, during the meantime, the daily irrigation of the parts with either the douche of Scanzoni or a gallon of infusion of hops or hydrastus canadensis, applied with the Davison or Mattison self-syringe, with the uterus in position, will ordinarily in time accomplish what I have in vain expected from the seemingly more energetic treatment with the caustic applications, so generally recommended by the best works upon gynæcology. My success in the management of nearly all the inflammatory affections of this organ has been much greater, as I use less frequently the acid nitrate of mercury, potass. com. calce., nitrate of silver, and kindred means, and adopt the agencies less heroic.—*Cincinnati Medical News.*

*A Modification of the Operation for Phymosis.*—Dr. Harrison Allen (*Medical Times*) recommends the following method:

Having first slit up the prepuce upon a grooved director—thus permitting the penis to assume its normal position to the sheath, and the true relations between it and the foreskin to be accurately determined—the operator takes a straight needle, of moderate size, and, arming it with a single strand of well annealed silver wire, transfixes both layers of the foreskin about a fourth of an inch in advance of the corona. Before pushing the needle through, that portion of the foreskin in front of the needle is snipped off with the scissors—sufficient tissue being reserved to bring the edges together—when a twisted suture is effected. The needle is next inserted at a point midway to the frænum, and the same procedure repeated as in the first instance. In consequence of the redundency of the skin about the frænum, the division of the mucous layer is best effected at that point before that of the skin. The scalpel is better than the scissors for this purpose. The skin should be removed by the latter instrument after transfixion. Two sutures introduced upon the opposite side, in the manner already given, complete the operation. Should the spaces between the wires gape, they may be approximated by silk threads. These may be removed within twenty-four hours. The silver wires should be retained a day longer.

It will at once be seen that the retraction of the skin-layer is prevented by transfixing the two layers at the same point before the removal of the integument. This, indeed, is the gist of the whole matter. The slitting of the prepuce, instead of being a mere expedient to remove the pressure from a threatened glans, or to explore for a concealed chancre—uses to which it is commonly restricted—becomes a stage of the operation of circumcision. Moreover, it enables the operator to dispense with the use of the fenestrated forceps.

This operation has been performed by myself and others fourteen times, and has given entire satisfaction in every instance.

*Prevention of Miscarriage by Hypodermic Injections of Morphia* (*American Journal of the Medical Sciences*).—Dr. A. B. Isham, of Cincinnati, after detailing three cases of success obtained by this plan, says:

"All the advocates of opium, with the exception of Tyler Smith, Byford, and Tanner, advise its administration per rectum. This is probably because the irritable stomach so common in cases of threatened miscarriage may not tolerate so nauseous a medicine as opium. In the manner of administration may have been the small measure of success. It is well known that medicaments introduced into the rectum act in a very uncertain manner; and it will be readily seen how opium or its alkaloids, given *per orem* to a patient vomiting and retching, might fail to avert a miscarriage. Just where these means have failed, and will fail again, is where the hypodermic injections would prove invaluable.

"It is useless to enjoin quiet. The patient is not likely to be quiet, suffering the pains incident to uterine expulsive efforts, her mind perturbed by fears for her own welfare and that of her child. Too often, too, the fruit of conception is regarded as an incumbrance, which it would be happiness to lose, and constant activity is one of the most popular recipes to the attainment of such end. Cold applications, leeching and blood-letting, would prove in most instances a waste of precious time; besides, at this day, such practice would be esteemed a barbarity, and not without some reason.

"The prime elements of danger to the life of the fœtus are—*first*, muscular contractions of the uterus, and *second*, hemorrhage. The latter most commonly results from the operation of the former; but it matters nothing as to the many and various causes tending to produce these phenomena. Considerations of safety imperatively demand a speedy arrest of uterine muscular action and hemorrhage, securing which, we may go about investigating and correcting the causes with comparative leisure.

"The use of morphia by hypodermic injection is the most speedy and certain means we possess of effecting such desiderata. It will do even more. Its influence spreads over the brain, and at the same time that it suspends contraction of the uterine muscular fibres it insures quiet and rest to the whole system, even against the patient's will. It is applicable to any case of threatened abortion, and I believe that its general use would render success at least equal, if not the rule, instead of the exception, as now announced by obstetricians. Of course I am aware that there are occasional individuals who do not well bear the exhibition of opium in any form, but I hold with Byford (Theory and Practice of Obstetrics, p. 171): 'I do not forget, in thus speaking freely about the use of opium, that peculiarities in some persons make it almost useless; yet I remember the unreliability of other remedies in these cases, and risk such disagreements.'"

*On the Indications against Version in Shoulder Presentations.* Dr. A. Pinard, in his *These d' Agregation*, defines version by external and internal manipulation, and then goes on to describe the method of operation in breech presentation. He then comes to the contra-indications which result (1) from the non-dilatation of the orifice; (2) from the too deep impaction of the fœtus; (3) from a too great narrowness of the pelvis. Amongst the causes which oppose themselves to the dilatation of the orifice and prevent the accomplishment of version, the writer quotes cancer and fibrous tumours of the neck of the uterus. The writer then quotes a certain number of cases in which the too deep impaction of the fœtus prevented version. The retraction of the uterus, the result of fruitless attempts at version or of taking oxytocic medicines, is one of the most formal counter-indications against pelvic version. The author afterwards examines how the accoucheur should proceed in narrowness of the pelvis when there is breech presentation. In pelves which are constricted, but still show an extent of 7 to 9 centimetres in their promonto-pubic diameter, version must be performed. But when the diameter is under 7 centimetres it becomes a question what is to be done. M. Pinard separates these pelves into two categories. In the first he places those which measure from 5 to 6 centimetres and a half; in the second, those which measure less than 5 centimetres. In the pelves of the first category, M. Pinard admits that version is sometimes possible but extremely difficult. It is only after having bruised, torn, lacerated, even perforated the genital canal, that the cephalic extremity is extracted. If we leave out of the question the labour and fatigue of the accoucheur, and only look to the mother and child, what is the result? The child is crushed to pieces, the mother is exhausted and weakened, and, notwithstanding all the skill of the operator, such disorganization of the genital parts may come on that, as a rule, death will be the consequence. The author then cites a series of cases in support of his views, and adopts the following conclusions: In pelves which measure from 5 to 6½ centimetres, if it be ascertained that the child is dead, version is still the only operation to which recourse can be had. But if the infant be living, version should be rejected, and recourse had to another operation. Pelvic version is impracticable in contraction under 5 centimetres. In the second part of his thesis, the author reviews the means by which version may be replaced: 1. When the neck is neither dilated nor dilatable; 2, when the fœtal portion is too deeply impacted; 3, when the uterus is constricted either by a prolonged labour or by the immoderate use of oxytocic drugs; 4, in extreme narrowness of the pelvis.—*London Medical Record*, Dec. 15, 1875. *Monthly Abstract of Medical Science.*

*Dr. Munde*, in New York *Academy of Medicine*, was of the opinion that no injury would follow, if the placenta after an abortion was permitted to remain in the uterus for a number of days after the expulsion of the fœtus, and by the introduction of a tampon and the use of ergot, etc., it could be, as a rule, found in the vagina within a very moderate length of time.

Dr. Hubbard remarked that for the last twenty years he had made it a rule to leave the placenta entirely alone in abortion occurring from one to four months. If hemorrhage is severe after the expulsion of the fœtus, use a tampon, and leave the case to take care of itself. He tampons in the following manner: Take about one ounce of pulverized alum, tie it up in a fine cambric handkerchief, and leave the string attached. Introduce this little bag into the vagina, and crowd it up against the neck of the uterus. Behind this he usually places a piece of soft sponge, which also has a string attached to it, and then he leaves the woman, feeling that she is perfectly safe. The next day this tampon is removed, and if hemorrhage occurs, another is introduced, and another day is allowed to pass. In a large number of cases the placenta will be found in the vagina quite certainly at the end of the second, probably by the end of the first day, and he believes this to be the safest practice. Interference he regarded as bad practice, provided the mouth of the uterous is not dilated sufficient to permit of the easy removal of the uterine contents.

For more than twenty years he has not introduced the hand into the cavity of the uterus for the purpose of removing a clot. All that is necsssary is to introduce the hand into the vagina, and two fingers into the cavity of the uterus, and break up the clot, when it will be rapidly expelled, if pressure is at the same time made over the uterus with the other hand. Now, if pain is present give ergot, for it will not act if pain is absent; and if pain is absent give opium and nux vomica, for the purpose of developing nerve-power. This was his method of treating this class of cases, and believed by Dr. Hubbard to be safe and effectual. If the placenta is adherent, another condition of affairs is present, and it may be necessary to proceed to its removal.

Dr. Garrish regarded the treatment by the immediate removal of the placenta in abortion as impracticable, for the reason that in many cases it is impossible to reach the cervix, so that the finger or any instrument can be introduced with safety for such purpose. His habit is to use a lump of alum tampon, with cotton or sponge, and permit the placenta to remain six or eight days if necessary. No serious consequences have ever attended this method of treatment. The vaginal surfaces are at the same time kept clean by the use of antiseptics.

Dr. Garrish was also of the opinion that chlorotorm administered during confinement predisposes to post-partum hemorrhage.

The Academy then adjourned.—*The Medical Record*.

*Bryant's Test Line—Its Diagnostic Value in Cases of Injury of Hip Joint.*—Dr. Thos. Bryant (*London Lancet*) says that for three years he has derived great benefit from the use of this line in the above class of cases. To obtain the line the patient is placed on a firm bed with his pelvis brought to a right angle with the spine, and his lower extremities slightly extended. A tape is now permitted to fall from the anterior superior spinous process of the ilium of one side to the horizontal plane of the body. A second tape measures the shorter distance from this vertical tape to the upper border of the great trochanter on the same side. Similar measurements are taken on the opposite side. The horizontal lines are compared, and if exactly alike, there is probably no lesion. At the hip joint, a shortening of the test line indicates a direct injury to the hip.—*Detroit Review*.

*Digitalis in Typhoid Fever*.—Dr. A. Patton, (*Cincinnati Lancet and Observer*) writes: "In typhoid fever there is high authority for the employment of digitalis. Dr. Hankel, a distinguished German writer, reports results of observations made in eighty cases of typhoid fever: 'Invariably there was a diminution of the fever and lowering of the pulse. It lessens delirium, and the pulse, when small, becomes fuller. It may be given to anæmic and depressed patients.' The attack is prolonged, so that the remedy is only indicated in cases of great danger. Dr. H. M. Jones, in the Dublin *Journal of Medicine*, 1874, says he has almost abandoned the use of alcohol in typhoid and typhus fevers, and substituted in its place digitalis, and his statistics demonstrate a greatly reduced mortality. He has treated a very large number of cases with it, and claims that it steadies the pulse, rendering it less compressible. He has observed its effects closely in two hundred cases, and has no doubt of its antipyretic effect. He regards it to be a wonderful cardiac tonic and stimulant, and not sedative. I have used the medicine with great caution in low forms of fever, and in a sufficient number of cases to become fully convinced of its great value in those severe forms of fever in which there is a small, quick pulse, delirium, and failing heart power, as indicated by the diminished intensity or entire suppression of the first sound of the heart, together with its alteration in duraton and quality. In these cases I have invariably found benefit from it, and greatly prefer it to alcoholic stimulants, or any other agent. For the heart softening, and for continued debility, which often occur in typhoid and typhus fevers, and is a fruitful source of fatality according to Louis and others, we want a remedy that will directly and certainly produce firm and decided contraction of the muscular tissues of the heart. That digitalis will accomplish this, we have a right to affirm from well-accredited facts.

*Pericarditis; Treatment by Means of Ice-Bags.*—A boy, fourteen years old, has been suffering from acute rheumatism. A complication of pericarditis and endocarditis resulted. The treatment pursued has been the application of ice-bags to the chest, over the region of the heart.

*Gallic Acid in Albuminuria following Scarlatina*—(*The American Practitioner*)—Dr. J. T. Jameson reports two cases of albuminuria occurring as a sequel of scarlet fever, and in which he employed gallic acid with marked success. In one case, a child æt. 6 years, caught cold during convalesence, and a day or two after the face became œdematous; there was pain in the head, and slight fever; the urine was quite bloody, and on testing in the usual manner presented considerable coagulation. The patient was put upon a saturated solution of gallic acid, a teaspoonful every two hours. In seven days the urine was free from albumen and copious in quantity, and the child seemed well, with the exception of debility, for which the muriated tincture of iron was prescribed. About ten days after this, in consequence of fresh exposure to cold, there was a slight relapse, the urine becoming again bloody and the face puffed; but on resuming the gallic acid for a few days these symptoms speedily subsided, and the recovery became permanent. In this case the gallic acid was administered unaccompanied by any other medicine, except an occasional dose of castor oil to regulate the action of the bowels.—*Medical Times.*

*Plugging the Nasal Cavities.*—Dr. T. H. Jewett describes (Philadelphia *Med. and Surg. Reporter*) the following simple plug in nasal hemorrhage: Roll up a lock of cotton into a cylinder an inch or an inch and a half in length; tie a strong thread to the middle of the roll; bring the two ends of the roll together, and then opening the nasal orifice, pass the middle or the folded part of the roll into the nostril; next with the blunt end of a lead-pencil press in the cotton roll slowly, along the floor of the nose, one inch or more and rest. If the blood passes down into the throat, you may be sure the bleeding spot is behind the roll; so push in your roll further, and the blood will cease to pass behind. Then, holding on to the string, pass some loose cotton into the nostril and push it down to the plug. The cotton will swell with the moisture and arrest the hemorrhage. In a day or two the natural secretions of the nasal surfaces will loosen the plug, and it may be easily loosened by the string.—*Jour. Mat. Med.*

*Anæmia*—(*The Lancet*).—Dr. Julius Polluck calls attention to that form of anæmia which is met with in young unmarried women, and is usually associated with some disorder of the catamenial function. Here lies chiefly upon steel to effect a cure; but if the tongue is coated and the digestion much impaired, the more astringent forms of iron, such as the sulphate or perchloride, are often not tolerated at first; and the ammonia citrate, the mistura ferri comp., or the ferrum redactum, will be the best to begin with. In a large number of cases he has found nothing so successful as a combination of the ammonia-citrate of iron and rhubarb in suitable doses, with equal parts of some bitter infusion and peppermint water. Sometimes the addition of two or three grains of the carbonate of ammonium seems to be useful. He makes rather a point of the rhubarb, although it is so disagreeable to take, believing it to assist the action of the steel, especially when the stomach is out of order. If the patient is very nervous, ten grains of the bromide of potassium may be added with advantage to each dose of the mixture. If the rhubarb in the mixture does not act enough upon the bowels, it will be necessary to prescribe some aperient pill to be taken at bedtime. Preparations containing aloes are of service, and may be combined with steel. Pepsine is often useful with the meals. The diet should be light and simple; beer had better be avoided in most cases, and a glaas or two of light claret may take its place with advantage. Claret is certainly better than port, although that wine is so often recommended. A moderate amount of exercise out of doors, when the weather permits, should be insisted upon, but anything like fatigue must be avoided. A tepid bath in the morning, and a rub down afterwards with a rough towel, is a good thing. In a few weeks, more or less, the steel and rhubarb mixture may be left off, and fifteen drops of the solution of perchloride of iron given after each meal in a wineglass of water.—*Medical News.*

*Solution of Bromine as Dressing.*—A valuable stimulating application is obtained by dissolving two drachms of bromine in a pint of water. The use of bromine in a dilute form is rather a novel method, and seems to offer some advantages.

*Nitric Acid as a Caustic in Uterine Practice and its Superiority as such to Nitrate of Silver.*—Nitric acid is the caustic which, of all others, is the best adapted for use in cases of chronic inflammatory disease of the os and cervix uteri, resulting in erosion or ulceration. Nitrate of silver is inefficient, and requires frequent reapplication, to atone for its defects both in degree and in the nature of its action. Nitric acid, on the other hand, acts as a caustic in these cases with certainty, and neither does too much nor too little. Its application is productive of little or no pain; and, when it has once been properly applied in some cases, no further speculum examination is required, such reliance may be placed upon its effects. If an exaimnation be made, which is always better, it need only be after an interval of a month, and then the acid may be applied again to any spot which appears to require it. The resulting sore has a very strong tendency to heal, and does so partly by contraction and partly by fresh formation of mucous membrane, which is not cicatrical in appearance. The contraction is greater than follows the application of any other caustic, and is the very thing required to insure the permanence of the cure. The contraction in cases of cervical catarrh is only contraction to a healthy size of the canal, provided the acid is used with proper care. The peculiar lasting and permanent action of nitric acid enables us to do away with the repeated speculum-examinations, so distasteful to both patient and surgeon, and gives the latter a feeling of confidence of success which he cannot have with any other caustic. The use of nitric acid, common as it is in other diseases, is referred to by very few writers, and is entirely omitted by most of our standard authors upon diseases of women, all of whom recommend nitrate of silver, or mention its use as the usual practice.—*Obstet. Jour.*

*Pills for Obstinate Neuralgia.*—The *Bordeaux Medical* gives the following formula for obstinate neuralgia, especially ileo-lumbar neuralgia: Valerianate of ammonia and quinine, each thirty grains. Make into twenty pills, and take from two to ten of them each day, increasing one pill per diem. After taking these pills for ten days suspend their use for five days.—*The Doctor.*

*A Certain Cure for Rheumatism.*—Judging from his article in the *Weiner Medizinische Presse*, Dr. Franz Heller is an enthusiast in the administration of caustic ammonia in rheumatism. For several years he has been a sufferer from severe muscular rheumatism in the right shoulder; he had taken all the common anti-rheumatic remedies with but little alleviation, when he began to reason that in rheumatism, as in gout, there may be a uric acid diathesis; he thought that liquor ammoniæ, on account of its rapid volatilization, would be the remedy most readily absorbed, and the most prompt in action. In almost the same moment, in which he took one drop, diluted with water, he felt a complete relief from the pain which had lasted for ten hours; he was now able to move freely the arm which, an instant before, he could scarcely bear to have touched. The remedy, he claims, has proved a positive cure in all recent cases of muscular rheumatism which have fallen under his observation; he cites numerous cases in which relief, as instantaneous as his own, was experienced.

He also observed its effects in several cases of acute articular rheumatism, in two of which six drops sufficed to subdue the pain and swelling within a period of twenty-four hours.

In one case of chronic rheumatism of a finger joint, which had lasted for over half a year, the simple administration of the ammonia completely dispelled the inflammation and pain in the joint within two days.

He then discusses the mode of action of his remedy. "If we consider an excessive acidity as the cause of the rheumatism, we can scarcely claim in the cases in which one drop will instantaneously relieve the pain in recent rheumatism, that that one drop was sufficient to counteract the effects of the excess of uric or according to Fuller) lactic acid.

"Nothing remains, therefore, but for us to seek for the sourca of rheumatism in a morbid nervous activity induced by disturbances of nutrition, and to believe that the ammonia acts as a nervine directly upon the nerves."

After the cure of one attack of rheumatism, our object should be to put the patient in such a condition as to prevent their recurrence. This, the writer thinks, can be done by building up the general system, and thus diminishing the nervous excitability.—*Clinic*, January 1, 1876.

*Typhoid Fever Probably caused by Infected Milk.*—Some time ago an article appeared in the London *Lancet*, showing that it is highly probable that typhoid fever may be spread by milk exposed to the emanations of typhoid fever patients. As further evidence in the same direction, I communicate the following:

In the month of August last, a young man, sick of typhoid fever, was brought to his father's house from Philadelpnia. The fever has prevailed at this house up to this date, with intervals of a few weeks between some of the cases, five in all, of which one died on November 22d.

On November 26th, I was called to my first case outside of this family, and from that date I have seen about twenty-five cases, occurring in about twenty different families, and spread along a narrow tract two miles long.

*Every one of the families had been supplied with milk obtained at the first mentioned place.* There had been no epidemic before the case was brought from the city, and there has not been a single case in the neighborhood where other milk was used.

On investigation, it was found that the washing of this sick family who sold milk was done at the spring-house where the milk was kept prepared for sale. The clothing of the sick and of the one who died was washed in the same boiler as were the pans and cans in which the milk was kept and served. The clothing lying around before being washed may have been another source of infection. The sale of this milk was stopped, and there has not been a new case for about two weeks.—*Med. and Surg. Reporter*, January, 1876.

*Iodide of Starch as an Antidote to Poison.*—Bellein (*Revue Medical*) is of the opinion that the combination of iodine and starch may prove an antidote to quite a number of poisons, with many of which it forms insoluble compounds, or compounds which, if soluble, are no longer poisonous. It is said to be particularly useful in cases of poisoning by strychnia, the result of a mixture of the poison with the iodide of starch being an exceedingly insoluble substance.—*The Laboratory.*

*Treatment of Orchitis.*—George N. Monette, M. D., (*American Practioner*) writes: "The following is the treatment which I have found uniformly successful in the different varieties of orchitis—syphilitic, gonorrhœal, and traumatic: In the first variety, where the body of the testicle and the epididymis are involved, the disease yields readily to specific medication. I usually give one-sixteenth of a grain of corrosive sublimate, with ten grains of iodide of potassium, every four hours, and use locally cantharidal vesication and the suspensory bandage. If pain in the course of the cord is severe, morphia hyperdermically or by the mouth. A marked reduction in the size of the inflamed organ will frequently be observed in from twelve to twenty hours, and the improvement is generally permanent.

"In gonorrhœal orchitis, I trust entirely to cantharidal vesication; the benefit is generally evident within two days at farthest. As in the syphilitic variety, morphia may be given if the pain demands it.

"Traumatic orchitis, which is comparatively rare, should be treated with cold water dressing until the swelling subsides somewhat, and then a cantharidal blister.

"In using a blister I seldom permit complete vesication to occur because of the severity of the suffering, and difficulty in dress-the blistered surface; but if the induration be persistent, then the entire organ should be completely blistered, using afterward castor oil dressing. After the vesication has healed, or even sometimes before, the bandage is used, first applying lint or charpie saturated with castor oil. After the bandage is care-ully and completetely applied, on account of cleanliness envelop, the whole with oil silk.

"I think this plan of treatmont much superior to any other which I have had the opportunity of observing, and it is believed it will prove quite as satisfactory in the practice of others as it has in mine."

*Treatment of Dysentery with Salycilic Acid.*—Cases of acute dysentery have been treated satisfactorily by means of doses of thirty grains of salycilic acid administered three times a day. The only objection to the remedy appears to be a slight irritation of the stomach, but this is not sufficient to cause emesis.

*Hip Disease — Its Treatment.*—Mr. Annandale (*Edinburgh Medical Journal*, December, 1875) gives the following summary of the views on this subject:

1. When suppuration and disorganization of the textures of the hip joint continue unrelieved by ordinary treatment, excision of the head of the femur is a proper and justifiable proceeding, if the patient's health is in a fair condition.

2. The operation is more successful in children than in adults.

3. In successful cases of the operation, the results, as regards the usefulness of the limb and joint, are superior to those which follow a natural cure.

4. Superficial or limited acetabular disease does not interfere with the performance and good results of excision of the head of the femur.

5. Even when the acetabulum is much involved, or pelvic suppuration exists, it is important to afford a free escape to the pus by the removal of the head, neck and great trochanter of the femur.

6. When the acetabulum is extensively diseased, it, together with the head and neck of the femur, should be removed, if the patient's condition admits of the operation.

7. It has not yet been accurately decided what is the earliest stage of the disease in which the operation is justifiable, although most agree that hitherto the operation has on the whole been delayed too long.—*Detroit Review.*

*Chloral Suppositories.*—The production of chloral suppository containing a sufficient proportion of this drug to cause sleep has heretofore been deemed impossible. Mr. H. Mayet, in the *Druggists' Circular*, has, however, devised the following formula, by which he manages to get forty-five grains of chloral in each suppository:

R. Ol. theobromæ........................gr. xxv.
Cetacei..................................
Pul. chloral.............................. aa gr. xlv.

For one suppository.

These suppositories are of good consistence, and may easily be put into use.—*Medical and Surgical Reporter.*

*Tinea Decalvans.*—B. F. Frishe, M.D., (*Louisville Medical News*) writes: On the 14th of February last I was called in by Mrs. W. to see her little boy, who she said was troubled with falling of the hair. I immediately recognized it to be a case of tinea decalvans. The bald spot on the crown of the head was about the size of a silver quarter, and daily increasing in extent. His mother had first noticed it a week before. Otherwise the little fellow was perfectly healthy, being only three years old, yet weighing something over forty pounds. I directed the head to be thoroughly washed, and then I applied pure carbolic acid to the entire diseased surface. This turned the skin a bright-red color, but did not blister. On the 18th I ordered the following compound:

| | | |
|---|---|---|
| R. | Tinct. cantharides............................ | aa drachm j. |
| | Sulphurous acid................................ | |
| | Sulphate of quinia............................. | gr. xv. |
| | Burnett's cocoaine............................. | oz. iv. |

The bald spot was well rubbed with this twice daily. At the end of two weeks an application of sulphurous acid was made. One week subsequently hair was observed springing up all over the place. The use of the hair tonic was still continued. Five weeks from the time the case came under my treatment the formerly diseased surface was completely covered with fine glossy hair fully half an inch in length. Indeed, except on very close inspection, it was impossible to notice any difference between that part of the scalp which had been and that which had not been affected.

What is remarkable in this case is the exceedingly short time of its cure. I would recommend the treatment to others, confident that they will find it as efficacious as I did.

*Salicylic Acid for Offensiveness of Breath and Expectoration.*—Dr. Da Costa. *Medical and Surgical Reporter*, prescribes salicylic acid, five grains, dissolved by means of a drachm of glycerine in half ounce of water, taken three times a day, in cases where the breath or expectoration are offensive. If internal administration does not accomplish the desired result, it can be used with the atomizer in a solution of similar strength.—*American Prectitioner.*

*Rupturing the Membranes.*—In a multipara with a normal pelvis and head presentation, I generally rupture the membranes at the earliest opportunity afforded me. I know that we can do no harm, as the parts yield very readily; but care must be taken, should the head be above the superior strait, that nothing but the head should entirely cover the os. It is a custom with some physicians to withdraw their fingers from the vagina the moment they have succeeded in evacuating the amniotic liquor; but this is injudicious practice, as at times there is so much fluid that the umbilical cord, or a hand or foot, is washed down with the first gush of water; and if these are not replaced at once, you have either a weeping family, or a difficult case to contend with. It is, therefore, a good rule, never, in such cases, to rupture the membranes until the head dips deep enough into the excavation.

The above remarks we find in a discussion before the Baltimore Medical Society by Dr. Seldner His precautions against withdrawing the fingers when the membranes are ruptured may be good, but show the practice of rupturing the membranes thus to be of doubtful propriety. The presence of the finger, while it might detect the prolapse of the cord, or the coming down of a hand or foot, could not prevent the occurrence. To rupture the membranes when the head is above the superior strait, even though the os be well dilated, should never be done except some special reason exists for interference.

*Catheterism in Laceration of the Urethra* (*The Lancet*).—Mr. Teevan reports two cases of retention of urine from laceration of the urethra, and remarks that if a laceration or false passage existed in the floor of the deep portion of the urethra, a curved metal catheter ought to be passed, as it could be made to hug the roof of the urethra, and so avoid slipping into the *cul-de-sac* in the floor. If, on the contrary, the laceration were in the roof, a straight elastic catheter ought to be introduced, as it always tended, when passed, to keep to the floor of the urethra, and would thus escape becoming locked in any rent or roof of the canal.—*Medical Times.*

*An Improved Method of Obtaining Support for Fractured Bones of the Extremities* (*New York Medical Journal*, September, 1875). —Dr. S. Wackerhagen uses the following method in treating fractures of the long bones :

After replacing the fragments as accurately as possible (extension being maintained by assistants), the limb is smoothly bandaged with cotton wadding, prepared in the form of an ordinary roller ; a flannel bandage spread with dry plaster of Paris, and rolled, is now soaked in warm water (to which are generally added about two fluid ounces of saturated solution of sulphate of potassium), and applied to the limb, over the wadding, by circular and reversed turns. One layer of the flannel applied in this way is amply sufficient for support.

When we wish to inspect the point of fracture, the dressing, which is only about an an eighth of an inch thick, is easily cut through by a pair of curved scissors.

If it be desired to employ lateral splints, the dressing should be cut in the median line of the anterior and posterior surfaces. If antero-posterior support is preferred, it should be cut through the lateral surfaces. The splints should now be varnished on their inner and outer surfaces with shellac, or this preparation may be applied to the outer surface before romoval.

The shellac seems to permeate the dressing sufficiently to increase the strength of the splint, and at the same time renders it slightly flexible instead of brittle, as is the case when plaster of Paris is used alone.—*Medical Times.*

*Electricity in Diseases of the Skin.*—Dr. Stone, in *Pacific Medcal Journal*, recommends electricity in affections of the skin, Musquito bites, urticaria, and the distressing irritation from rhus poisoning may all be relieved by passing a current over the affected parts. In the poisoning from rhus he saturates two pieces of cloth with salt water and passed them with both poles, one in each hand, over the part affected, the curent being very weak. The burning sensation is returned in ten minutes, and a repetition of the process in three or four hours will be followed by a speedy recovery.

*Digitalis.*—Dr. Patton (*Cincinnati Lancet and Observer*) writes: "As to the diuretic powers of the medicine, my observations correspond with the statements of Dr. McLean, who wrote his book sixty years ago, that it never directly stimulates the renal organs to increased activity, but in dropsy acts by powerfully exciting the absorbent system, which causes the serous effusions to be taken up, and the kidneys being the natural outlet for the superabundant fluids of the system, there is established a physiological diuresis. When the kidneys are torpid, they will require stimulation by special diuretics, or a vicarious action will probably be established in the bowels or salivary glands. When there is no dropsical effusion, not the slightest diuretic action of the digitalis has been observed in a single case.

"Digitalis should be given uncombined with other medical agents. I usually give it diluted with pure water. Acids of all kinds have the effect to render digitalis inert. This was asserted seventy-five years ago, and I find it to be a fact. Iron should not be used with it, nor should aconite, as the first is, to some extent, chemically incompatible, while aconite is antagonistic in its action. So is veratrum veride. In fact no medicine should be given at the same time, or at a shorter interval than two hours from digitalis.

*Chloroform in the Surgery of Infants.*—Dr. Bergeron, of Paris, (*London Obstetrical Journal,*) from his personal experience upon this subject concludes that

1. Chloroform in the infant is endowed with an almost absolute harmlessness.

2. The child, not having come to the age of reason, nor feeling any moral emotion, suffers from no apprehension of the dangers to which it may be exposed, nor experiences the apnœa which produces so much terror, and is, the author imagines, a most important cause of death supervening suddenly during the administration of chloroform.

3. Chloroform may be administered to the infant from the first day of its birth.—*Amer. Med. Weekly.*

*A New Use for Carbolic Acid—Opening Abscesses without Pain.* Dr. Bergonzini, of Bologna—(*Revista di Bologna,* 1875.)—Put on the skin for three or five minutes, a solution of two parts of carbolic acid to one part of glycerine. If the skin be previously inflamed (as is ordinarily the case in abscesses following great inflammation) a too protrated contact of the anæsthesiant liquid must be avoided. Dr. Bergonzini, who has used this very simple means with success, thinks that it can be utilized in autoplastic operations as well as in superficial neuralgia.—*Chicago Medical Journal.*

*Chlorate of Potassa in Vaso-Paralytic Diarrhœa.*—With this term Dr. Bonfigli designates the diarrhœa which attends the latter stages of diseases of the nervous system. This diarrhœa is characterized by frequent alvine and serous evacuations, without abdominal tenderness or other digestive disorder, and the autopsy does not reveal the slightest trace of anatomical alteration. The author reports forty-five observations, from which it results that chlorate of potassa is one of the most useful remedies in this kind of diarrhœa. He insists, however, that the remedy should be continued a certain length of time, and that it is necessary to give it in doses of 30—150 grs. in twenty-four hours. In cases of cachexia combined with much nervous depression, the remedy acts slowly, and is ineffectual when there is any active disturbance of the intestinal mucous membrane.—*Lyon Med.* from *Gaz. Med. Prov. Ven.*

*Caustics in the Treatment of Diseases of the Cervix*—Gilt—(*British Med. Jour.*, December, 1875.)—The author sums up the results of his observations as follows: 1st. In comparatively recent cases of endocervicitis, nitrate of silver, tincture of iodine or carbolic acid suffices. 2nd. Chronic cases of endocervicitis had best be treated by acid nitrate of mercury, or nitric acid. 3rd. Hyperchronic endocervicitis, with considerable cervical hypertrophy, requires potassa fusa cum calce, or some strong acid. He thinks Dr. Braithwaite is too general in his recommendation of nitrate of silver.—*Chicago Med. Jour.*

*Grindelia Robusta—Its Medical Value.*—Dr. H. M. Fiske (*Pacific Medical Journal*, August, 1875) says that in all cases of iritis he has obtained the happiest results from the use of Grindelia Robusta. It matters not what the cause of the iritis may be—whether gout, rheumatism, scrofula or violence. He gives internally a teaspoonful of the fluid extract every four hours, and externally to the diseased eye a solution of one part of four of water. In asthma he has found its administration of considerable benefit. It controls the distressing nausea and retching attendent upon all stages of cancer of the stomach better than any other remedy he was able to find.

Dr. John E. Cowe (*Louisville Medical News*, February 12th, 1876,) reports eleven cases of bronchitis, complicated with asthma, in which the use of Grindelia was followed by the happiest effects.—*Detroit Review.*

*Bromide of Lithium.* — Dr. Levy (*Bull. Gen. de Therap. Journal Nerv. and Ment. Diseases*, January, 1876) gives the results of some studies on this salt:

(1) It has no action upon the muscular system, while the bromide of potassium has a distinct influence upon it. (2) Bromide of lithium acts in general, more energetically and more rapidly on the cord and sensory nerves than the bromide of potassium. (3) The loss of sensibility, commencing in the nerves, may propagate itself in a longer or shorter time to the cord or even the brain.

In gout, bromide of lithium has a slight action, but it does not act by diminution of the quantity of uric acid. Bromide of lithium has a marked sedative action on the cerebro-spinal axis. It modifies favorably various neuroses, especially epilepsy. In this respect it is more active than bromide of potassium. Moreover, it does not affect the heart as does bromide of potassium. In gout the dose of bromide of lithium is seven grains. In nervous disorders, as hysteria, insomnia, etc., the dose may be three grains. In epilepsy the dose may be increased to half a drachm. *Detroit Review.*

*Ulcerated Nipples.*—M. Legroux advises the following treatment: Spread with a camel-hair brush a layer of elastic collodoin around the nipple, in a radius of an inch or more; a piece of gold-beater's skin should then be placed over the nipple and collodion, taking care to make a few holes with a pin over the part of the gold-beater's skin which covers the nipple, so as to allow the milk to ooze through. No collodion should be spread on the nipple itself, as some pain might thereby be occasioned. By the rapid evaporation of the ether the collodion dries up, and the gold-beater's skin adheres. The nipple is then more or less pressed down by the latter, which in drying becomes tense. When the child is to be nursed, the end of the nipple should be wetted with a little water. The gold-beater's akin which covers it becomes soft and supple, allows the nipple to swell, and protects the ulcers and fissures from the strain of suction. The mother or wet-nurse thus suffers no pain, and the ulcers heal in a few days.—*Lancet*, Dec. 11, from *Annales de Gynecologie*, Nov., 1875.

*Bromide of Camphor—Its Therapeutic Value.*—M. Pathault (*Gaz. des Hopitaux*, *Journal Nervous and Mental Diseases*, January, 1876,) gives a collection of clinical observations respecting the value of this drug. Great benefit was obtained from its use in cases of chorea, dilirium tremens aud hysteria in ali its phases, hysteria-epilepsy, epilepsy, neuralgia, nocturnal pollutions, and various affections of the genito urinary tract. It is best administered in capsules.—*Detroit Review.*

*Ergot of Rye as an Antipyretic.*—Hayem (*Rev. de Therap.; The Clinic*,) says: "The writer tried ergot in cases of enteric fever, with the object of lowering the temperature. The results were very satisfactory, and its employment in this disease seems to M. Hayem preferable to that of quinia or digitalis. Under ergot there is a more rapid defervescence; and at the period of the acme, instead of there being a rise in the temperature chart, a plateau is obtained. In some cases in which the ergot was only given during the day, the evening temperature was not so high as the morning. The dose varied from thirty to fifty grains in twenty-four hours."

*What Vaseline Is.*—Vaseline, a product of petroleum, is a pale yellow, translucent, slightly fluorescent semi-solid, melting at about 37° C. Specific gravity 0.840 at 55° C. It is inodorous, non-volatile at ordinary temperatures, but distills with slight decomposition under pressure. It is insoluble in water, slightly soluble in alcohol, freely in ether, and miscible in all proportions when melted with fixed or volatile oils. It mixes in all proportions with glycerine of the ordinary strength, but the mixture is destroyed by the addition of water. Hydrochloric acid and liquor potassæ are without action upon it.

Vaseline was first brought before the notice of the public by Professor Otis, of New York, who states that the article is largely used as a basis for ointments, and by himself for lubricating surgical instruments, and so facilitating their introduction into the passages.—*Medical and Surgical Reporter.*

*Chloroform as a Preservative Agent.*—Mr. J. Schafer, of New Orleans, La., sends to the *Druggists' Circular* the report of an interesting case of suicide by chloroform:

A young lady committed suicide at Pass Christian, Miss., by swallowing about four fluid ounces of chloroform. She died at half-past four o'clock on Saturday afternoon, the 5th of the month, two hours and a half after taking the poison. The remains were sent to the friends of the deceased in New Orleans, and at the end of one hundred and forty-seven hours continued to look as natural as they did but half an hour after death.

The remarkable case of a corpse being preserved without change for over six days, in a damp and warm climate like that of New Orleans, attracted the attention of the medical men who sat as a jury at the inquest. The known action of chloroform was assumed to be the cause of the unusual preservation of the body.—*Medical and Surgical Reporter.*

*Ergot for the Intestinal Hemorrhage of Typhus.*—Dr. Plagge, strongly recommends the extract of ergot, in combination with tincture of opium, with cherry laurel water as a vehicle, and given every half hour, in the treatment of hemorrhage from the bowels occurring in typhus fever. He has found the ergot much better than ice and astringents.—*American Practitioner.*

*Chlorine Water in the Treatment of the Injuries Received from Rabid Animals.*—Nearly half a century ago chlorine water as a lotion was proposed for destroying the virus in the treatment of wounds caused by the bite of rabid animals. Wendelstadt reported a case in 1809 which he had successfully treated in this manner.

Later, Semmola and Schoemberg strongly advocated it; the latter is said to have treated nineteen cases with good success.

In view of these and other statements found in the books, the editor of the *Journal de Chimie Medicale* suggests the utility of experiments in this direction, for the purpose of determining to what extent such treatment is efficacious. If, as is claimed by some, cauterization does not always succeed, it is important that some further information should be obtained in regard to the treatment of accidents, which almost always result in death.—*The Laboratory.*

*Intestinal Hœmorrhage in Typhoid Fever.*—Dr. Th. Plagge (*Memorabilien*, xx, 11,) says: "The author has noticed the application of ice upon the abdomen, the administration of liq. ferri, alum, catechu, kino, tannic acid, acetate of lead, etc., to be very inefficient in hæmorrhages of the kind named above, and he is, therefore, very happy in having found a powerful and efficient remedy in ergot. His patient, a laborer, aged thirty-two, was in the second week of typhoid fever; delirious; face flushed; tongue very dry and furred; involuntary dejections. During the night of Nov. 28, he had discharged almost a pint of pure blood, his face was pale, his feet cold, pulse very feeble and frequent. He was ordered to take sherry and a mixtnre of extract of ergot with tincture of opium in cherry water. During that day he had three more bloody stools, but then the patient rallied and rapidly recovered."

*Treatment of Burns.*—In the treatment of burns, when of a superficial character, a preparation consisting of two parts of collodion and one of olive-oil has been found to be very efficacious. When the burn is of an extensive character, gasoline proves of decided benefit. The advantage of gasoline is, that it is of the right consistence, and does not become rancid.

*Salicylic Acid for Foul Breath and Offensive Expectoration.*—Prof. De Costa (*Phila. Med. and Surg. Reporter*, Feb. 1876,) says; "The following prescription, used as a mouth wash and gargle has yielded much satisfaction in the above troubles:

"R. Sodii boratis, acidi salicylici, *aa*, grs. x; glycerine, dr. j; aquæ, ad oz. j. M."

The professor recommends this combination in cases of foul breath arising from almost any cause whatever, as from fetid bronchitis, from disordered stomach, or indigestions with bad breaths from pulmonary abscess or cancer, etc., etc. Five grains, three times a day, were given; the purification of the breath was accomplished satisfactorily; the expectoration was improved in odor, but not abolished.

*How to Make Raw Meat Palatable to Invalids.*—The following receipt for this purpose has been given by Ivon: Raw meat (from the loin), 250 grammes (8.7 oz.); shelled sweet almonds, 75 grammes (2.6 ounces); shelled bitter almonds, 5 grammes (.17 ounce); white sugar, 80 grammes (2.8 ounces); these substances to be beaten together in a marble mortar to a uniform pulp, and the fibers to be separated by a strainer. The pulp, which has a rosy hue and a very agreeable taste, does not at all remind one of meat, and may be kept fresh for a considerable time, even in summer, in a dry, cool place. Yolk of egg may be added to it. From this pulp, or directly from the above substances, an emulsion may be prepared which will be rendered still more nutritious by the addition of milk. Lailler prefers the following preparation: Dried raw meat, 100 grammes (3.5 ounces); sugar, 40 grammes (1.4 ounces); wine, 20 grammes (.7 ounce); tincture of cinnamon, 3 grammes (.1 ounce). It is a kind of electuary, very agreeable to the palate.—*Industrie-Blatter.*

*Parametrttis and Peri-Metritis.*—Schroeder (Diseases Female Sexual Organs, page 45) says that parametritis is a connective tissue phlegmon, which is due to an infection with septic material; hence, that it is common in the puerperal state, but at other times is tolerably rare, and that perimetritis is a partial peritonitis, which may be, and frequently is, induced by the most diverse causes.—*Detroit Review.*

*Salicylic Acid in Acute Rheumatism* (*The Boston Medical and Surgical Journal*, February 24, 1876.)—Dr. Charles P. Putnam reports the case of a child, five years of age, who had been suffering from acute rheumatism for five days, who had a pulse of 130, a temperature of 102.7 degrees. Salicylic acid was given in five-grain doses every hour when the patient was awake. After the first three doses there seemed some improvement; after the seventh dose she went to sleep, and only took two more doses during an entire night. In the morning she was markedly better, and forty-eight hours after treatment began the temperature was 99.9 degrees.

*Treatment of Scrofulous Sores or Runnings.*—McLennan (*Med. Times and Gazette*, 1876,) says: "For the past twelve or thirteen years he has treated the above named troubles with corrosive-sublimate in whisky. Three grains of this salt, put into a pint bottle of whisky, is the strength of the solution used. A rag dipped in this preparation twice or three times a day and laid on the sore and continued till the sores are all dried up, is all that is needed. In no case in which this treatment has had a fair trial, has it failed."

*Dressing for Burns.*—Dr. Q. C. Smith, of Cloverdale, Cal., (*Pacfiic Medical and Surgical Journal*,) says: "Mix sub-nitrate of bismuth with pure honey till it forms a thick paste, and spread it over the burn and contiguous parts plentifully, and cover with cotton wool batting, and bind closely. Remove the dressing in three or five days by soaking in water, and re-apply same remedy. The reporter considers, after trying many kinds of dressing, that this dressing is the best.

*Jervia—A New Alkaloid in Veratrum Viride.*—In the *Amer. Jour. of Pharmacy* for October, 1875, Charles Bullock describes the alkaloid *jervia*, known for a number of years as existing in the root of veratrum album, but now found by him in the American species, *viride*. Its physiological properties are described by Prof. H. C. Wood, Jr., as producing general weakness, without vomiting or purging, lowering arterial pressure and slowing of the pulse, profuse salivation and finally convulsions. The character of the convulsions is very peculiar and very constant.

